# The imidazoacridinone C-1311 induces p53-dependent senescence or p53-independent apoptosis and sensitizes cancer cells to radiation

**DOI:** 10.18632/oncotarget.16102

**Published:** 2017-03-10

**Authors:** Anna Skwarska, Shaliny Ramachandran, Grzegorz Dobrynin, Katarzyna B. Leszczynska, Ester M. Hammond

**Affiliations:** ^1^ Cancer Research UK and Medical Research Council Oxford Institute for Radiation Oncology, Department of Oncology, The University of Oxford, Oxford, UK; ^2^ Department of Pharmaceutical Technology and Biochemistry, Chemical Faculty, Gdańsk University of Technology, Gdańsk, Poland

**Keywords:** p53, C-1311/Symadex, radiation, senescence, apoptosis

## Abstract

C-1311 is a small molecule, which has shown promise in a number of pre-clinical and clinical studies. However, the biological response to C-1311 exposure is complicated and has been reported to involve a number of cell fates. Here, we investigated the molecular signaling which determines the response to C-1311 in both cancer and non-cancer cell lines. For the first time we demonstrate that the tumor suppressor, p53 plays a key role in cell fate determination after C-1311 treatment. In the presence of wild-type p53, cells exposed to C-1311 entered senescence. In contrast, cells lines without functional p53 underwent mitotic catastrophe and apoptosis. C-1311 also induced autophagy in a non-p53-dependent manner. Cells in hypoxic conditions also responded to C-1311 in a p53-dependent manner, suggesting that our observations are physiologically relevant. Most importantly, we show that C-1311 can be effectively combined with radiation to improve the radiosensitivity of a panel of cancer cell lines. Together, our data suggest that C-1311 warrants further clinical testing in combination with radiotherapy for the treatment of solid tumors.

## INTRODUCTION

The imidazoacridinone C-1311 (Symadex™), is a multi-purpose therapeutic agent which has been tested clinically. As an anti-tumor agent, C-1311 has been tested in a phase I study for patients with advanced solid tumors and was found to have an acceptable toxicity profile [1]. C-1311 is a unique small molecule with a number of diverse functions. The most characterized function of C-1311 is as a DNA-damaging intercalator/alkylator, which exerts an anti-tumor effect in part through inhibition of topoisomerase II. The inhibition of topoisomerase II by C-1311 leads to the formation of cleavable DNA-topoisomerase II complexes and subsequent DNA strand breaks [2]. However, more recent studies have described additional roles for C-1311; the inhibition of tumor angiogenesis through the targeting of the HIF-1α/VEGF pathway [3], inactivation of the human cytochrome P450 1A2 and 3A4 isoenzymes [4], and inhibition of receptor kinases, most notably the FMS-like tyrosine kinase FLT3 [5].

Perhaps, in part due to the multiple attributes of this agent, the cellular response to C-1311 is equally complex. A number of studies have described the induction of mitotic defects, apoptosis, autophagy and senescence in response to C-1311 [6, 7]. However, the factors that govern cell fate after C-1311 exposure have not been reported. How cancer cells and indeed the surrounding normal cells respond to a chemotherapeutic agent is critical to their use in combination with additional therapies. In response to DNA damage, cancer cells may temporarily arrest the cell cycle in order to repair DNA damage, or if this is not possible, initiate lethal programs to prevent the propagation of genomic abnormalities [8]. Mitotic catastrophe results from the premature entry of cells into mitosis before the completion of DNA repair [9]. These cells become enlarged, contain multiple micronuclei and eventually die by apoptosis or necrosis [10]. Whereas apoptotic or necrotic cell death results in the clearance of cancer cells, cells which cease to proliferate and enter a senescent state remain viable and retain their metabolic potential [11]. Activation of senescence is often achieved by low doses of chemotherapeutics, which allows for the long-term growth arrest of malignant cells while diminishing the toxic effects to normal tissue [12]. However, senescent cells may also secrete growth promoting factors and pro-inflammatory cytokines, which can stimulate the proliferation of surrounding cancer cells. This in turn raises the question as to what extent chemotherapy-induced senescence is a desired outcome of cancer treatment [8, 13]. Interestingly, alternative DNA alkylating agents such as cisplatin, or topoisomerase II inhibitors such as mitoxantrone, have been shown to have p53-dependent effects including the induction of senescence [14–17]. Mitoxantrone was shown to induce a senescence-associated secretory phenotype (SASP) in several prostate cancer cell lines, and this was increased in the absence of functional p53 [18].

Here, using human colon cancer cell lines we provide a molecular analysis of the response to C-1311 exposure. Cells with wild-type p53 underwent p53-mediated G_2_ phase arrest and ultimately senescence. In contrast, cells lacking p53, despite an initial arrest in G_2_ entered mitosis, underwent mitotic catastrophe and apoptosis. In addition, we provide the first evidence that C-1311 increases the radiosensitivity of a number of cell lines and therefore has a potential role as a radiosensitizer.

## RESULTS

### In response to C-1311, p53-null cells undergo apoptosis

**Figure 1:**
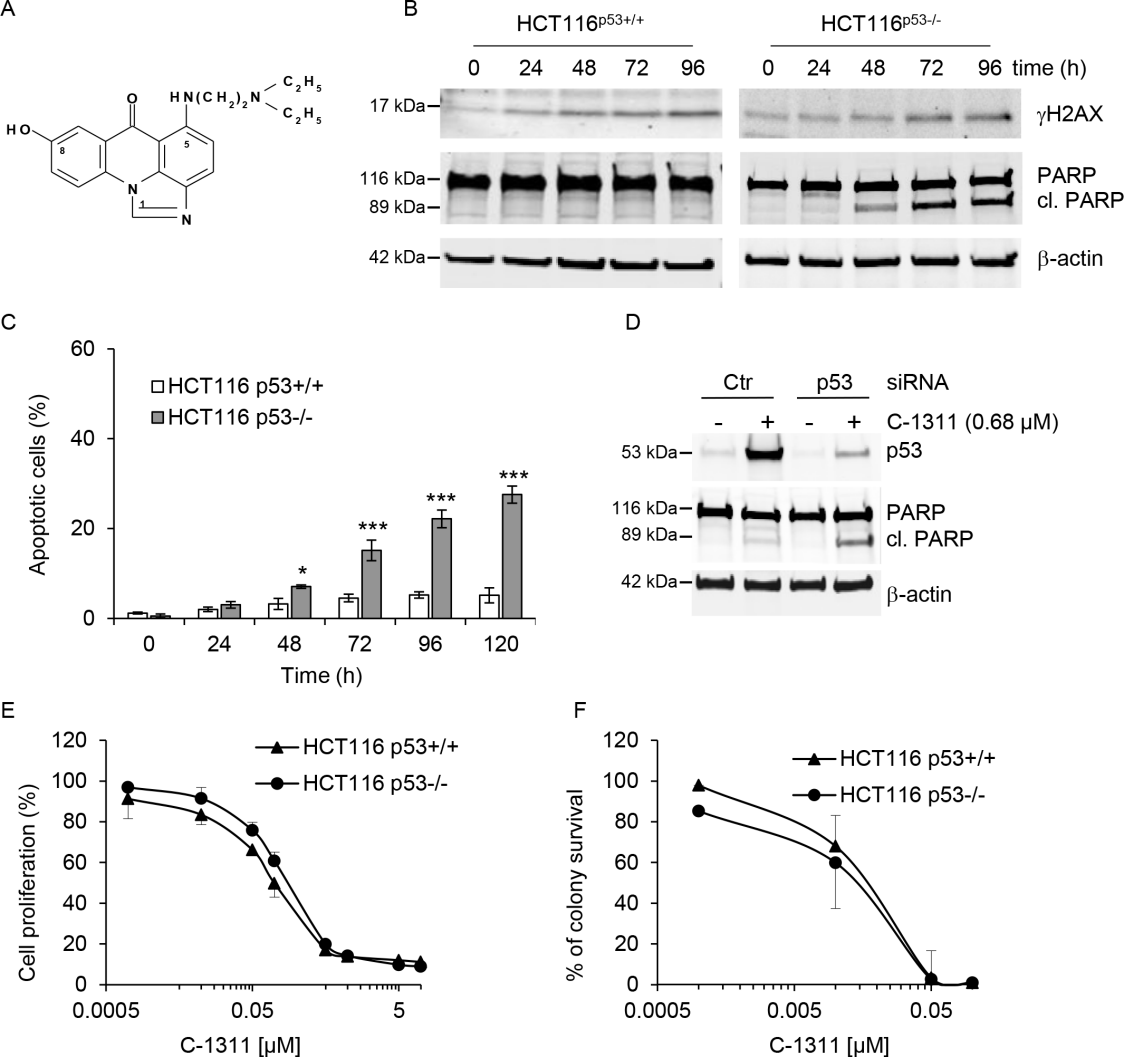
C-1311 induces apoptosis in cells lacking p53 (**A**) Chemical structure of imidazoacridinone C-1311. (**B**) HCT116 p53^+/+^ and p53^−/−^ cells were exposed to C-1311 (0.68 μM and 0.64 μM, respectively) for the times indicated. Western blotting was then carried out for γH2AX and PARP. β-actin was used as a loading control. (**C**) HCT116 p53^+/+^ and p53^−/−^ cells were exposed to C-1311 as in (B), stained with DAPI and nuclear morphology was examined using fluorescence microscopy. Cells with shrunken, intensely stained and fragmented nuclei were scored as apoptotic. The data are presented as mean ± SD, *n* = 3. *P < 0.05, **P < 0.01, ***P < 0.001 *vs* control group. (**D**) RKO cells were treated with p53 or scrambled (CTR) siRNA for 24 h, and then siRNA was removed and cells were exposed to C-1311 (0.68 μM) for 72 h. Western blotting was carried out for p53, PARP and β-actin as a loading control. (**E**) HCT116 p53^+/+^ and p53^−/−^ cells were exposed to C-1311 for 72 h. Proliferation rates were determined by cell counting. Results are a mean ± SD, *n* = 3. (**F**) HCT116 p53^+/+^ and p53^−/−^ cells were exposed to C-1311 and colonies were counted 14 days after treatment to determine survival fraction. Results are a mean ± SD, *n* = 3.

To assess the effect of the p53 tumor suppressor on the biological response to C-1311 (Figure 1A), we used the genetically matched pair of human colon carcinoma cell lines, HCT116^p53+/+^ and HCT116^p53−/−^ [19]. Each cell line was exposed to C-1311 and western blotting was carried out (Figure 1B). Unsurprisingly, histone H2AX was phosphorylated (γH2AX) in response to C-1311 exposure in both cell lines, which is suggestive of DNA damage but could also indicate replication stress and/or senescence [20–22]. However, most strikingly, we observed PARP cleavage, indicating C-1311-induced apoptosis, specifically in the HCT116^p53−/−^ cells. Given this surprising finding of apoptosis induced in response to a DNA damaging agent only in the absence of p53, we sought to verify our finding with additional assays. We found that in the HCT116^p53−/−^ cell line, C-1311 induced an accumulation of cells with apoptotic features such as condensed, intensely stained chromatin and apoptotic bodies. In contrast, analysis of nuclear morphology revealed that C-1311 did not significantly induce cell death in the p53-proficient HCT116 cells (Figure 1C). FACS analysis using Annexin V-FITC in conjunction with propidium iodide staining further confirmed the induction of apoptosis specifically in the p53-deficient HCT116 cells by C-1311 (Supplementary Figure 1). To ensure that our observations were not restricted to the HCT116 cell line, we used siRNA to deplete p53 from an alternative colorectal cancer line, RKO. In agreement with the HCT116 cell lines, we found that C-1311 exposure led to increased PARP cleavage when p53 was depleted from RKO cells (Figure 1D). Together, these data demonstrated that C-1311 triggered apoptotic cell death specifically in cells lacking p53. This led us to question if p53 status would impact proliferation or overall survival after exposure to C-1311. We found that, C-1311 decreased proliferation of both HCT116 cell lines in a dose-dependent manner; however, no difference in sensitivity was observed between wild-type and p53-null cells (Figure 1E). IC_80_ values were calculated to be 0.68 and 0.64 μM for HCT116^p53+/+^ and p53^−/−^ cells, respectively. Subsequent clonogenic survival assays also demonstrated that the loss of p53 did not affect long-term viability after exposure to C-1311 (Figure 1F). These data demonstrate that although in the absence of p53, C-1311 exposure leads to apoptosis, the effects on proliferation/cell viability are independent of p53.

### C-1311 has a significant p53-dependent impact on cell cycle progression

**Figure 2:**
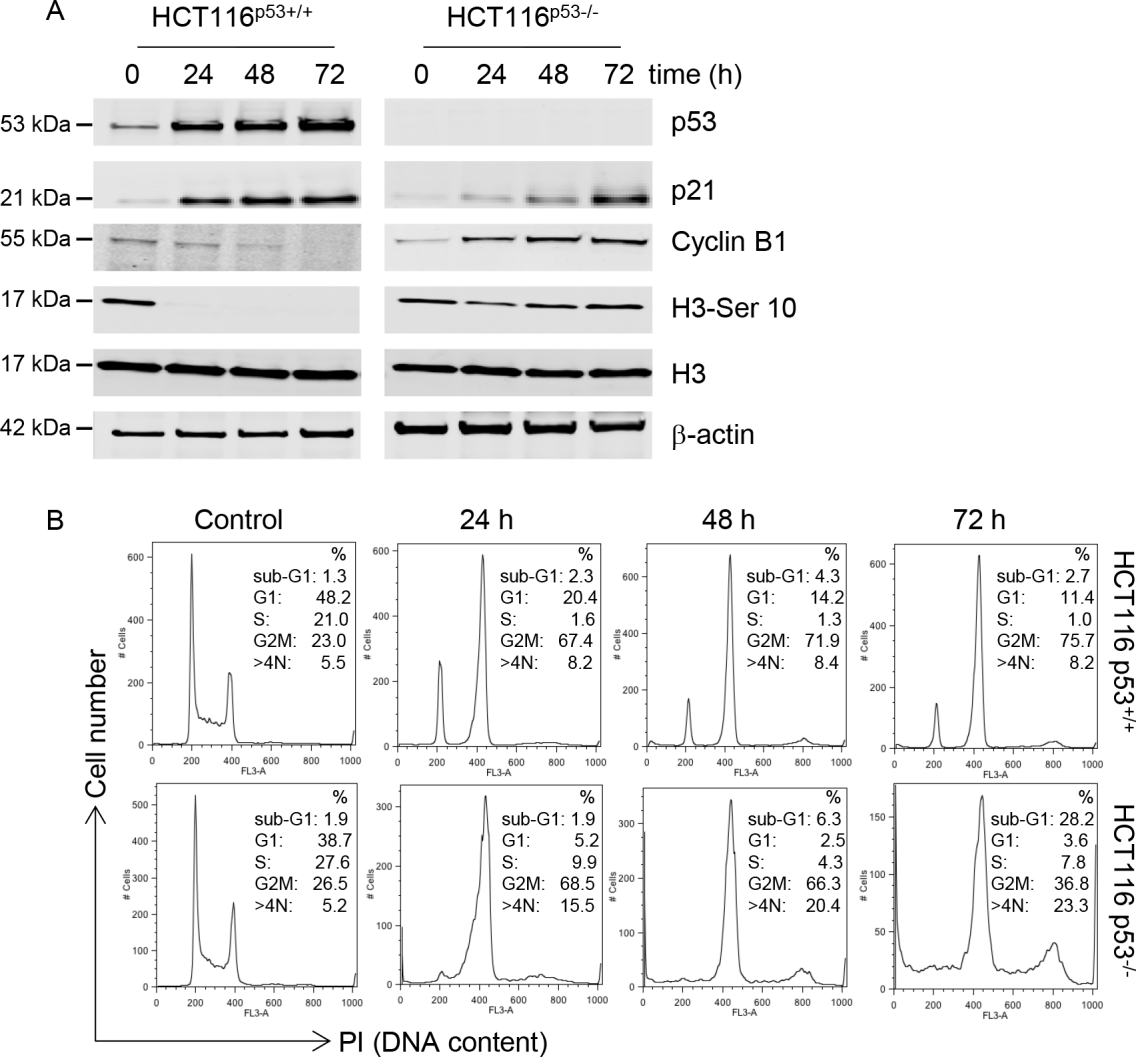
C-1311 has a p53-dependent effect on cell cycle progression (**A**) HCT116 p53^+/+^ and p53^−/−^ cells were exposed to C-1311 (0.68 μM and 0.64 μM, respectively) for the times indicated. Western blotting was then carried out for p53, p21, cyclin B1, histone H3 and H3-Ser10. β-actin was used as a loading control. (**B**) HCT116 p53^+/+^ and p53^−/−^ cells were exposed to C-1311 as in (A) and DNA content was determined following PI staining by FACS. Histograms are representative of three independent experiments.

The C-1311-induced DNA damage, detected by phosphorylation of H2AX, in HCT116^p53+/+^ cells was accompanied by elevated levels of p53 and its direct transcriptional target, p21 (Figure 2A). In contrast, in the HCT116^p53−/−^ cells, p21 activation was comparatively delayed. we analyzed the levels of cyclin B1 (expressed in late S, G2 and M phase) and histone H3 phosphorylated at Serine 10 (elevated during mitosis). In agreement with the observation that p53 can repress transcription of *cyclin B1*, thus contributing to G_2_ arrest [23], p53 wild-type cells treated with C-1311 showed a progressive decrease in cyclin B1 levels accompanied by a complete loss of H3-Ser10 (Figure 2A). In contrast, p53-deficient cells exposed to C-1311 showed significantly elevated cyclin B1 levels, and after a slight decrease, a strong and prolonged accumulation of H3-Ser10 (Figure 2A). These differences in key cell cycle regulators in HCT116^p53+/+^ cells compared to the p53-null cell line led us to question the p53-dependent effect of C-1311 on cell cycle progression. In the presence of p53, cells treated with C-1311 (0.68 μM) for 24–72 h accumulated in the G_2_/M phase of the cell cycle, which was accompanied by an almost complete loss of S-phase cells and progressive disappearance of cells in G_1_ (Figure 2B and Supplementary Figure 2A). The p53-deficient cells exposed to C-1311 (0.64 μM) showed a more rapid accumulation in the G_2_/M phase and, in contrast to HCT116^p53+/+^ cells, following prolonged drug exposure, p53^−/−^ cells progressed from a diploid to a polyploid state (cells with > 4N DNA content). In addition, there was a profound increase in cell death (cells with a sub-G_1_ DNA content) in the HCT116^p53−/−^ line but not the p53 proficient cells, which support our previous findings (Figure 1). To further explore the G_2_ arrest observed after C-1311 exposure we determined the mitotic index for both p53 wild type and deficient cells. In HCT116^p53+/+^ cells exposed to C-1311 for 24 h, the fraction of mitotic cells dropped 7 fold and the addition of nocodazole did not decrease the mitotic fraction further (Supplementary Figure 2B). In contrast, the mitotic index of p53^−/−^ cells remained unaffected following C-1311 treatment, and then doubled after the addition of nocodazole (Supplementary Figure 2B). These data demonstrate that although in response to C-1311, both p53 proficient and deficient HCT116 cells accumulated in G_2_, the cells lacking p53 were able to progress into mitosis.

Similarly to HCT116^p53+/+^ cells, when RKO cells were treated with C-1311, they arrested in G_1_ and G_2_ (Supplementary Figure 2C), and this was accompanied by elevated levels of p53 and p21, as well as decreased expression of cyclin B1 and H3-Ser10 (Supplementary Figure 2D). In contrast, when p53 was depleted using siRNA, RKO cells exposed to C-1311 rapidly arrested in the G_2_ phase, and corresponding to the lack of p53, expressed reduced p21 compared to the control RKO cells. Importantly, in p53-depleted RKO cells, as in HCT116^p53−/−^ cells, the G_2_ arrest was not sufficient to prevent cells entering mitosis, as evidenced by elevated levels of cyclin B1 and H3-Ser10 after 72 h of C-1311 treatment as compared to control RKO cells (Supplementary Figure 2D).

To verify that C-1311 had the same effects on the cell cycle in conditions that are physiologically relevant to solid tumors, we repeated our analysis in hypoxic conditions. This is of further importance as we have shown previously that C-1311 inhibits hypoxia inducible factor-1α (HIF-1α) activity, which can in turn impact cell cycle progression [3]. We found that under hypoxic conditions (1% O_2_), C-1311 had similar effects on clonogenic potential in both p53^−/−^ and p53^+/+^ HCT116 cells as observed in normoxia (Supplementary Figure 3A and 3B). Moreover, the C-1311 p53-dependent effects on the cell cycle observed in normoxia were also seen in hypoxic conditions (Supplementary Figure 3C–3F). These data suggest that the significant, p53-dependent effect of C-1311 on the cell cycle observed in normoxia, would also occur in cells residing in the hypoxic fraction of solid tumors.

### C-1311 induces mitotic catastrophe in p53-deficient cells

**Figure 3:**
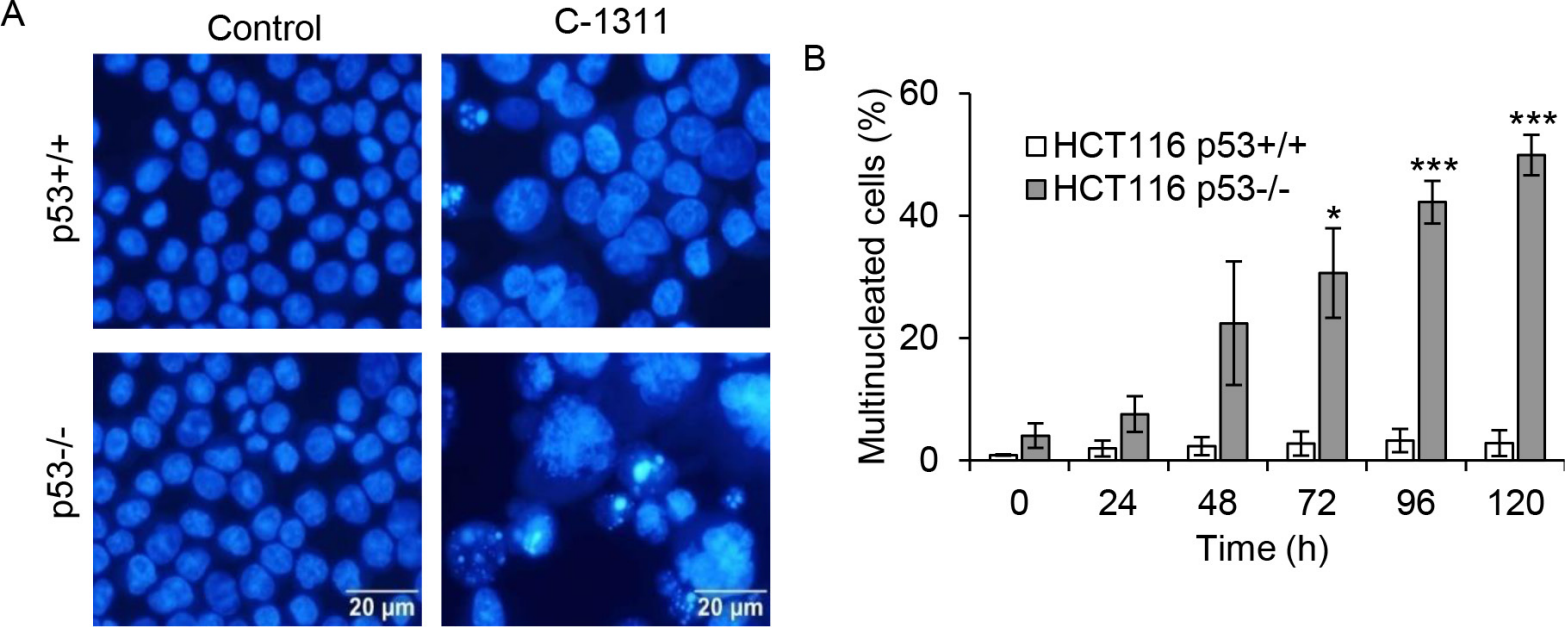
C-1311 induces mitotic catastrophe in p53-deficient cells (**A**) HCT116 p53^+/+^ and p53^−/−^ cells were exposed to C-1311 (0.68 μM and 0.64 μM, respectively), stained with DAPI and nuclear morphology was analyzed by fluorescent microscopy. Cells containing evenly stained multiple small nuclei were scored as cells undergoing mitotic catastrophe. Representative photographs obtained after 120 h of C-1311 treatment. (**B**) Quantitation of the percentage of multinucleated cells. The data are presented as mean ± SD, *n* = 3. *P < 0.05, **P < 0.01, ***P < 0.001 *vs* control group.

The finding that following C-1311 exposure, HCT116^p53−/−^ cells, despite an initial arrest in G_2_, entered mitosis in the presence of DNA damage, prompted us to investigate the cellular consequences of this G_2_/M checkpoint deficiency. Several studies have demonstrated that, depending on the severity of DNA damage, premature/aberrant mitosis leads to mitotic catastrophe followed by cell death [10]. Fluorescent microscopy analysis of cells revealed that treatment of p53-negative HCT116 cells with C-1311 led to an early accumulation of cells displaying morphological changes characteristic of mitotic catastrophe (Figure 3A). After 48 h of C-1311 exposure, when the G_2_/M arrest peaked, over 20% of p53-deficient cells were multinucleated with completely or partially separated micronuclei and evenly stained chromatin (Figure 3B). The development of giant, multinucleated cells increased during prolonged drug treatment (to approximately 50% after 120 h), and as shown by FACS analysis, was accompanied by an increase in polyploid cells (> 4N DNA, Figure 2B), a known characteristic of cells undergoing mitotic catastrophe. In contrast to HCT116^p53−/−^ cells, only a non-statistically significant (< 4%) fraction of p53-positive cells became multinucleated after C-1311 treatment, even after prolonged (120 h) exposure (Figure 3B). The lack of mitotic catastrophe in HCT116^p53+/+^ cells exposed to C-1311 is consistent with our observation of a stable G2 arrest and the loss of mitotic markers such as cyclin B1 and H3-Ser10 (Figure 2A). These data highlight the p53-dependent cell cycle response to C-1311.

### C-1311 induces senescence in p53-proficient cells

**Figure 4:**
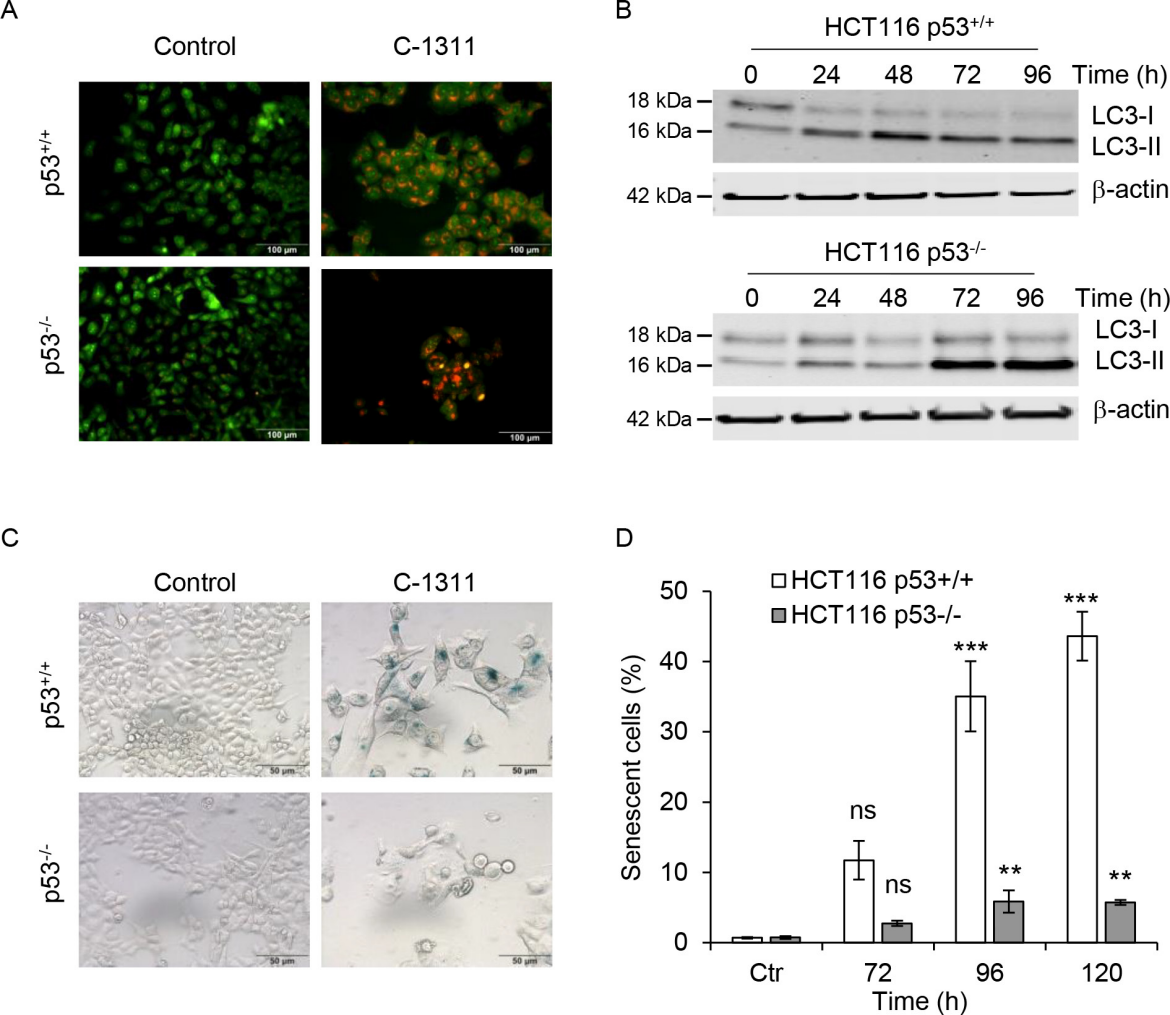
The p53 status determines cell ultimate biological response to C-1311 treatment (**A**) HCT116 p53^+/+^ and p53^−/−^ cells were exposed to C-1311 (0.68 μM and 0.64 μM, respectively) for 24 h, stained with acridine orange and analyzed by fluorescent microscopy. Acidic compartments characteristic for autophagy fluoresce bright red or orange-red, whereas nuclei and cytoplasm remain green. Representative image of three independent experiments. (**B**) Western blotting analysis of autophagic conversion of LC3-I to LC3-II. Cells were treated as in (A) for the times indicated. β-actin was use as a loading control. (**C** and **D**) HCT116 p53^+/+^ and p53^−/−^ cells were treated as in (A) for the times indicated, and stained for SA-β-gal activity characteristic of senescence. (C) Representative images for cells treated with C-1311 for 120 h. (D) Quantitation of the percentage of senescent cells. The data are presented as mean ± SD, *n* = 3. *P < 0.05, **P < 0.01, ***P < 0.001 *vs* control group.

As C-1311 appears cytotoxic independently of p53 status, despite inducing apoptosis specifically in p53-null cells, we questioned the fate of the p53-proficient cells after C-1311 treatment. C-1311 has been previously shown to induce autophagy in A549 and H460 lung cancer cells (both wild-type p53) [7]. After 24 h of C-1311 treatment, we observed the accumulation of acidic vesicular organelles (AVOs) in HCT116^p53+/+^ and HCT116^p53−/−^ cells (Figure 4A). This qualitative assessment of autophagy was further confirmed by western blot analysis of the conversion of LC3-I protein to the lipidated form, LC3-II, which takes place during autophagy upon autophagosome formation [24]. Consistent with the induction of AVOs, from 24 h after exposure to C-1311, there was a substantial accumulation of LC3-II in both p53^+/+^ and p53^−/−^ HCT116 cells, which suggests that C-1311-induced autophagy is independent of p53 (Figure 4B).

It has been reported that the fate of cells undergoing mitotic catastrophe includes cell death by apoptosis or necrosis, however, senescence is also a possible outcome [10, 25–27]. As HCT116^p53+/+^ cells exposed to C-1311 avoid both apoptosis and mitotic catastrophe, we hypothesized that the decrease in clonogenic survival could be associated with increased senescence. Supportively, we found that in HCT116^p53+/+^ cells, within 72 h of C-1311 exposure, 10% of cells were enlarged, flattened and stained positively for SA-β-gal (Figure 4C and 4D), which is a characteristic of senescence [28]. The proportion of SA-β-gal-positive cells increased to approximately 40% after 120 h of C-1311 exposure. In contrast, only small numbers of senescent cells were found in HCT116^p53−/−^ cells even following prolonged drug treatment (Figure 4C and 4D). This suggests the model that in the presence of p53, cells exposed to C-1311-induced DNA damage enter senescence whilst those lacking p53 undergo mitotic catastrophe and apoptosis.

### C-1311 induces senescence in non-cancer cells

**Figure 5:**
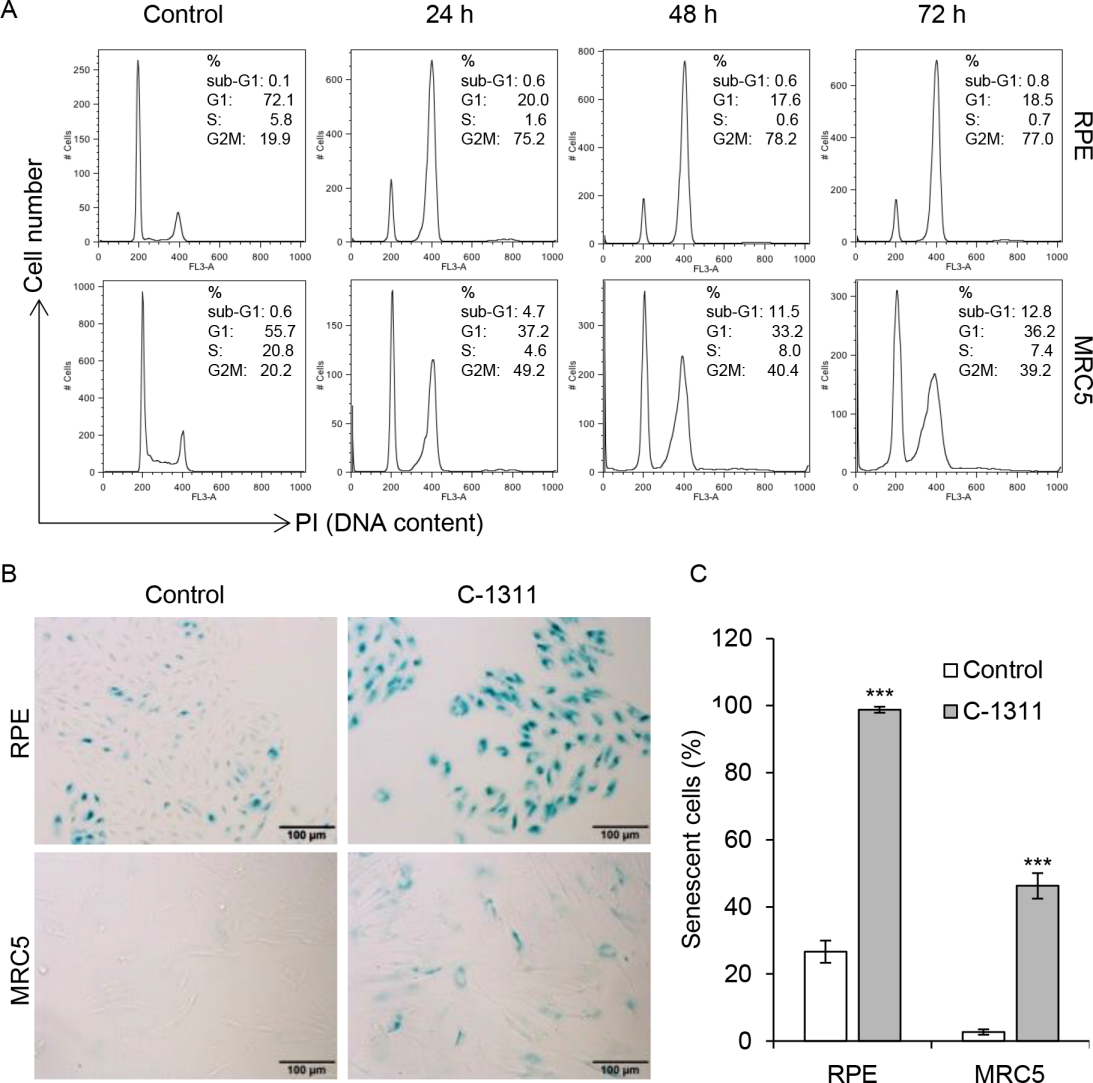
Increased senescence in non-cancer cells treated with C-1311 (**A**) Retinal pigment epithelial RPE cells and fetal lung fibroblast MRC-5 cells were treated with C-1311 (0.68 μM) for the times indicated and DNA content was determined following PI staining by FACS. Histograms are representative of three independent experiments. (**B** and **C**) RPE and MRC5 cells were exposed to C-1311 (0.68 μM) for 120 h, stained for SA-β-gal activity. (B) Representative images from bright-field microscope. (C) Quantitation of the percentage of senescent cells. The data are presented as mean ± SD, *n* = 3. ***P < 0.001 *vs* control group.

Next, we examined the effect of C-1311 on human retinal pigment epithelial (RPE) cells and human fetal lung MRC-5 fibroblasts. FACS analysis demonstrated that the majority of RPE cells (over 70%) arrested in the G_2_/M phase of the cell cycle (Figure 5A and Supplementary Figure 4A and 4B). Importantly, there was no evidence of an increased sub-G_1_ fraction and hence cell death. Similarly, MRC-5 cells treated with C-1311 accumulated in G_1_ as well as in G_2_M phase. The overall fraction of dead cells, although higher than in RPE cell line, did not exceed 10% of the total cell population after 72 h of treatment. Quantitative microscopic analyses indicated that after 120 h exposure to C-1311, all adherent RPE cells were enlarged and stained positively for SA-β-gal, suggesting the induction of senescence (Figure 5B). This phenomenon, although less profound, was also observed in MRC-5 cells; after 120 h of drug treatment over 40% of the MRC-5 cell population were senescent. These data again support the conclusion that one of the principal biological responses to C-1311 in p53-proficient cells is senescence and that this also occurs in non-cancer cells.

### C-1311 is an effective radiosensitizer

**Figure 6:**
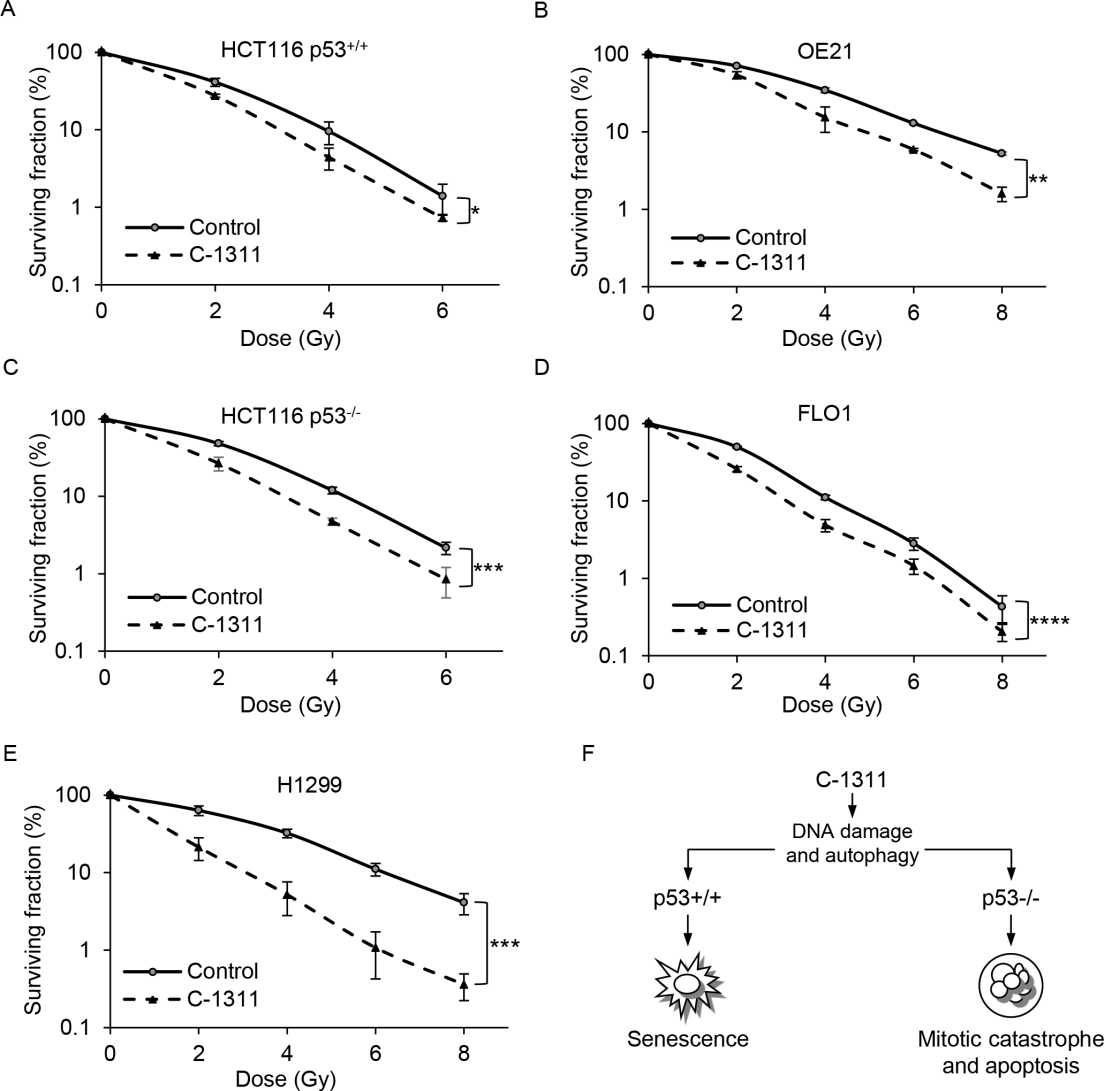
C-1311 increases sensitivity of cancer cells to ionizing radiation HCT116^p53+/+^ (**A**), OE21 (**B**), HCT116^p53−/−^ (**C**), FLO1 (**D**) and H1299 (**E**) cells were treated with C-1311 (0.1 μM) for 1 hour before exposure to a range of doses of ionizing radiation (0, 2, 4, 6 and 8 Gy). The medium was changed after 24 h and colonies were counted 10-14 days later. Results shown are mean ± SEM, *n* = 3. Significance: Two-way ANOVA test,*P < 0.05; ^*^P < 0.01; ^**^*P < 0.001; ^***^*P < 0.0001. (**F**) A model of p53-dependent response of cancer cells to C-1311 treatment.

DNA-damaging agents such as C-1311 are unlikely to be used as monotherapies and so determining their efficacy in combination with standard therapies is essential. It has been demonstrated that radiosensitivity depends in part on the phase of the cell cycle; whereas cells in S-phase are relatively radioresistant, cells in G_2_/M are more radiosensitive [29, 30]. As C-1311 induces a significant accumulation of cells in G_2_/M, we investigated whether the drug might also affect radiosensitivity. We extended our studies to include additional cell lines with mutated p53 (esophageal squamous carcinoma FLO1 and OE21 cells) or without p53 (non-small lung cancer H1299 cells). Cells were exposed to a low dose of C-1311 (0.1 μM) for 1 h before radiation treatment and clonogenic survival assays were carried out (Figure 6). The level of radiosensitization was determined by comparing the surviving fraction after 2 Gy (SF_2_), a dose that is typically used in clinical therapy. Although C-1311 significantly increased radiation-induced loss of viability in all the tested cell lines, the degree of radiosensitization was variable. The HCT116^p53+/+^ and p53-mutated OE21 cells were the least sensitized by C-1311 to radiation. The SF_2_ was reduced by C-1311 from 41.21% ± 4.9 in the control, to 27.51% ± 1.2 for HCT116^p53+/+^, and from 71.12% ± 1.4 to 53.99% for OE21 cells (Supplementary Table 1) (Figure 6A and 6B). An increase in C-1311 concentration (from 0.1 to 0.25 μM), appeared to result in greater radiosensitization in the OE21 cell line, suggesting a dose-dependent effect (Supplementary Figure 5). Upon combination of radiation with C-1311, the SF_2_ fraction dropped from 48.04% ± 2.9 to 26.64% ± 5.4 for HCT116^p53−/−^ (Figure 6C), and from 49.62% ± 1.3 to 25.82% ± 1.9 for FLO1 cells (Figure 6D). The p53-null H1299 cells were the most sensitive to radiation combined with C-1311 treatment; the SF_2_ decreased from 63.45% ± 9.0 to 21.28% ± 6.9 (Figure 6E). Together, these data suggest that C-1311 is an effective radiosensitizer and that this property does not appear to correlate with p53 status.

## DISCUSSION

Here, for the first time, we show that the cellular response to C-1311 treatment is largely determined by p53 status. In the presence of p53, cancer cells undergo senescence and this is also apparent in non-cancer cells. However, in the absence of p53, which is representative of the majority of human tumors, cells experience mitotic catastrophe and apoptosis (Figure 6F). Although our study is the first to demonstrate a p53-mediated response to C-1311, further support for this conclusion has been previously reported. A549, H460 and HepG2 cells, which all express wild-type p53, have been described as entering C-1311-induced senescence [7, 31], while HT29 and MOLT4, which have mutated p53, underwent mitotic catastrophe followed by cell death in response to C-1311 [6, 32]. These data are fully supportive of our model and confirm that the disparate reports in the literature have occurred due to a failure to consider p53 status.

The first cellular response to C-1311 exposure appears to be increased autophagy independently of p53 status (Figure 6F) [7]. It is not clear how or if, this increase in autophagy determines the subsequent p53-dependent effects. How p53 induces senescence in a predominantly G_2_ population is unclear and is unlikely to be entirely p21-dependent [33–35]. Further studies to investigate the transcriptional response to p53 after C-1311 are required to address this question.

Importantly, our studies are in agreement with recent reports questioning the long-held paradigm that tumors harboring wild-type p53 respond more favorably to DNA-damaging therapy due to the induction of apoptosis. Lozano and colleagues suggested that in breast cancer cells exposed to doxorubicin, wild-type p53 activity is paradoxically detrimental to chemotherapy response. Unlike mutant p53 tumors, p53 wild-type cancer cells can avoid aberrant mitosis by undergoing cell cycle arrest and senescence followed by expression of cytokines that can stimulate cell proliferation and tumor relapse [36]. In this regard new potential chemotherapeutics that induce mitotic catastrophe leading to apoptosis or necrosis in p53-deficient cells warrant particular attention [37]. Furthermore, since p53 may impair the apoptotic response to chemotherapy favoring cell cycle arrest and senescence, it has been proposed that this phenomenon could be exploited to protect normal cells from the toxic effects of chemotherapy [38]. Accordingly, given that non-cancer cells possess wild-type p53, its activation by DNA-damaging drugs should arrest their cell cycle without damaging them. In contrast, the p53-deficient cancer cells that enter mitosis in the presence of unrepaired DNA damage will be selectively eliminated by subsequent apoptosis/necrosis. Our studies show that C-1311, at the dose required to kill p53-deficient HCT116 cells, induced cell cycle arrest and senescence not only in p53-proficient HCT116 cells, but also in non-cancer RPE and MRC-5 cells. This in turn suggests that C-1311 has the potential to protect normal cells while attaining selective cytotoxicity towards cells lacking p53. However, the long-term consequences of C-1311-induced senescence and likely increased autophagy on normal cells is unclear and requires further study to determine if this would be detrimental.

Most importantly, we also show that C-1311 can be effectively combined with radiation to increase the radiosensitivity of a range of cancer cell lines. The increases in radiosensitivity observed did not appear to be entirely dependent on p53 status, suggesting the DNA-damage induced by C-1311 contributes to this effect. Although various factors have been reported to influence the radioresistance of cancer cells, one of the most important has been identified as the cell cycle status [30]. As mitotic cells are the most susceptible to ionizing radiation, the abrogation of the G_2_ checkpoint and cell progression into mitosis has emerged as an attractive therapeutic target to improve the efficacy of radiation therapy [37]. Therefore, it is possible that the use of C-1311 for prolonged periods of time before radiation, or during a course of fractionated radiotherapy, might increase the population of cells in mitosis in p53 null cells and increase radiosensitivity even further. C-1311 has been well tolerated in phase I clinical trials, and interestingly, has shown some promise in phase II in patients with breast cancers refractory to taxanes and anthracyclines [1, 39]. Our study strongly supports the further testing of C-1311 in combination with radiotherapy for the treatment of solid tumors.

## MATERIALS AND METHODS

### Cell lines and reagents

Human colorectal cancer HCT116^p53+/+^ and HCT116^p53−/−^ cells [19] were kindly provided by Prof. Bert Vogelstein (Johns Hopkins University, Baltimore, MD, USA) and were grown in DMEM (Sigma-Aldrich, UK). Human colorectal cancer RKO cells (p53^+/+^) and non-small cell lung cancer H1299 cells (p53^−/−^) were obtained from ATCC and were grown in DMEM. Human esophageal squamous carcinoma OE21 cells (p53-mutant) and human esophageal squamous adenocarcinoma FLO1 cells (p53-mutant) were from PHE culture collections and were cultured in RPMI and DMEM, respectively. Human retinal pigment epithelial RPE cells and human fetal lung fibroblast MRC-5 cells were a kind gift from Dr Geoff Higgins (Oxford) and were grown in DMEM/F12 and MEM, respectively. Cells were used within 20–30 population doublings. For each cancer cell line, culture medium was supplemented with 10% fetal bovine serum and 100 μg/ml streptomycin and 100 U/ml penicillin (Sigma-Aldrich). RPE and MRC-5 cells were grown in the presence of 10% FBS but without antibiotics. All cells were cultured in a humidified incubator at 37°C with 5% CO_2_. Cells were routinely tested for mycoplasma and found to be negative. All regents, unless stated otherwise, were from SIGMA-Aldrich (UK).

### Drug and radiation treatments

Imidazoacridinone C-1311 (Symadex™) was synthesized at the Department of Pharmaceutical Technology and Biochemistry, Gdańsk University of Technology, Poland, and was a kind gift from Prof. Jerzy Konopa (Symadex is also available from Sigma-Aldrich). C-1311, prepared as a stock solution at 5 mM in water and stored at −20°C, was diluted in culture medium to concentrations indicated in individual experiments. For the combination of C-1311 and irradiation, cells were seeded in 6-well plates in triplicates for each condition and 6–8 h later pre-treated with C-1311 for 1 h. Subsequently, cells were irradiated with γ-rays from a Cs-137 irradiator (GSR D1 Gamma-Service Medical GmbH, Germany; Dose rate 1.7 Gy/min) as described previously [40]. The medium was replaced with drug-free media after 24 h, and radiosensitizing effect of C-1311 was assessed by clonogenic assay as described below.

### FACS

Following drug treatment, cells were trypsinized and pooled with floating cells, washed twice with PBS, and fixed in 70% (v/v) ethanol overnight at −20°C. Next, cells were stained with PBS containing 20 μg/ml PI and 100 μg/ml RNase A. DNA content was analyzed by FACScan (Becton Dickinson, UK), and cell cycle distribution was determined using FlowJo software (Tree Star, USA).

### Proliferation assay

The effect of C-1311 on cell proliferation was measured using a ZB1 Coulter Counter. Cells were left to attach overnight and then treated with the drug for 72 h. After treatment, cells were trypsinized and counted. Cell survival was expressed as percentage of vehicle-treated control.

### Clonogenic survival assay

Cells were seeded in 6-well plates in a set of three for each treatment condition. Cells were exposed to C-1311 alone or in combination with irradiation as indicated, and colonies were allowed to grow for 10–14 days. Then, cells were stained with 2% crystal violet diluted in 50% methanol and 20% ethanol fixative mixture and counted. Plating efficiency for each treatment was calculated by dividing the numbers of colonies by the number of cells seeded. The surviving fraction was calculated by dividing plating efficiency for treatment by the plating efficiency for respective control, and expressed as a percentage.

### Immunoblotting

For Western blot analyses, cells were lysed in urea lysis buffer (8 M urea, 10 mM Tris-HCl, 0.1 mM β-mercaptoethanol). Lysates were sonicated and cellular debris were removed by centrifugation at 13 500 rpm at 4°C for 15 mins. Protein concentration was determined using NanoDrop spectrophotometer (Thermo Scientific, UK). Proteins (50 μg) were separated by SDS-PAGE and transferred to nitrocellulose membranes. The following antibodies were used: anti-p53 (DO-I, Santa Cruz Biotechnology), anti-p21 (#2947 Cell Signaling Technology), anti-cyclin B1 (#4138, Cell Signaling Technology), anti-phospho-histone H3 Ser 10 (#9701, Cell Signaling Technology), anti-histone H3 (#3638, Cell Signaling Technology), anti-PARP (#9542, Cell Signaling Technology), anti-LC3A/B (#4108, Cell Signaling Technology), anti-β-actin (C4, Santa Cruz Biotechnology). Immunoblots were developed using Li-COR Odyssey imaging system.

### Acridine orange staining of acidic vesicular organelles (AVOs)

Development of AVOs, which are the hallmark of maturation of autophagosomes during autophagy was visualized using acridine orange, as described previously [7]. Briefly, following drug treatment, cells were incubated for 15 minutes with medium containing 0.5 *μ*g/ml acridine orange. Next, cells were rinsed with PBS and immediately analyzed using a fluorescence microscope (Olympus BX60, Japan). Within AVOs, acridine orange forms aggregates that emit bright red fluorescence, whereas the cytoplasm remains bright green.

### Assessment of mitotic catastrophe and apoptosis

Following drug treatment, cells were trypsinized and pooled with floating cells, washed twice with PBS, cytospun onto microscopic slides, fixed and stained with 4′,6′-diamidino-2-phenylindole dihydrochloride (DAPI). Nuclear morphology was examined using a fluorescence microscope. Cells with shrunken, intensely stained and fragmented nuclei were scored as apoptotic. Cells containing evenly stained multiple small nuclei were scored as cells undergoing mitotic catastrophe. At least 100 cells per field of vision were counted for each sample in five random fields.

### Senescence associated-β-galactosidase (SA-β-gal) activity

SA-β-gal staining was performed using Senescence Detection Kit (#K320-250, BioVision Inc, UK) according to the manufacturer's instructions. Briefly, following continuous drug treatment, cells were washed twice with PBS, incubated with fixing solution for 15 mins, and after additional washing in PBS, stained with working solution containing 1 mg/ml of X-Gal. Senescent cells were identified as the blue-stained cells under bright-field microscopy (Olympus BX60 and Nikon Ti-E, Japan). At least 100 cells per field of vision were counted for each sample in five random fields.

### Statistical analysis

Statistical analysis was performed using GraphPad Prism 6 software (GraphPad Software Inc.). For the colony survival assays the 2-way ANOVA test was used. For all other analyses, unless stated otherwise, one-way ANOVA test was used, followed by Dunett test for each comparison. *P* values of less than 0.05 were considered as significant (*P < 0.05; ^*^P < 0.01; ^**^*P < 0.001; ^***^*P < 0.0001; ns non-significant).

## SUPPLEMENTARY MATERIALS FIGURES AND TABLES



## Abbreviations

FITC: fluorescein isothiocyanate
FLT3: FMS-like tyrosine kinase 3
HIF-1α-: hypoxia inducible factor 1 alpha
PBS: phosphate-buffered saline
SA-β-gal: senescence associated β-galactosidase
VEGF: vascular endothelial growth factor
X-Gal: 5-bromo-4-chloro-3-indolyl-β-D-galactopyranoside.
