# Supplementary material for: The imidazoacridinone C-1311 induces p53-dependent senescence or p53-independent apoptosis and sensitizes cancer cells to radiation
Source: Oncotarget. 2017 Mar 10;8(19):31187–98. doi: 10.18632/oncotarget.16102 (PMC5458200; doi:10.18632/oncotarget.16102)
Supplement: Supplementary file 1 [file oncotarget-08-31187-s001.pdf]

The imidazoacridinone C-1311 induces p53-dependent senescence or p53-independent apoptosis and sensitizes cancer cells to radiation

Supplementary Materials

MATERIALS AND METHODS

Dual Annexin V-FITC and propidium iodide staining

Following drug treatment cells were harvested, rinsed twice with PBS and stained with Annexin V-FITC and PI using Annexin V-FLOUS staining kit (Roche, Poland). Cells were analyzed with FACScan flow cytometer (Becton Dickinson, Poland) and quantified using WinMDI software.

Determination of mitotic index

Following C-1311 exposure for 24 h, cells were treated with nocodazole (150 nM) for 18 h, harvested by trypsinization and fixed in 70% ethanol overnight. The following day, cells were washed twice with PBS containing 1% FBS and stained with anti-histone H3 (H3-Ser10) antibody (#9701, Cell Signaling Technology) for 2 h. Cells were then washed with PBS and incubated with anti-rabbit antibody conjugated with Alexa Fluor 488 (ThermoFisher Scientific, UK) for 1 h followed by staining with PI solution (20 μg/ml PI, 100 μg/ml RNase A, in PBS). Samples were analyzed using a FACScan (Becton Dickinson, UK) and quantified with FlowJo software (Tree Star, USA).

Small interfering RNA (siRNA) transfection

Human p53-targeted and negative control siRNAs were purchased from Life Technologies. Cells were transfected with 50 nM p53- or control siRNA using Dharm-FECT1 reagent (Thermo Fisher Scientific) according to manufacturer's instructions. Cells were used 24 h later for subsequent experiments. siRNA sequences are available upon request.

Hypoxia treatment

Before hypoxia exposure, cells were seeded overnight to attach under normoxia (21% O_2). The next day, C-1311 was added and cells were immediately transferred into a H35 Hypoxystation (Don Whitley Scientific, UK) set to 1% O_2 and 37°C and incubated as indicated. Cells were lyzed inside Hypoxystation and subjected to western blotting.

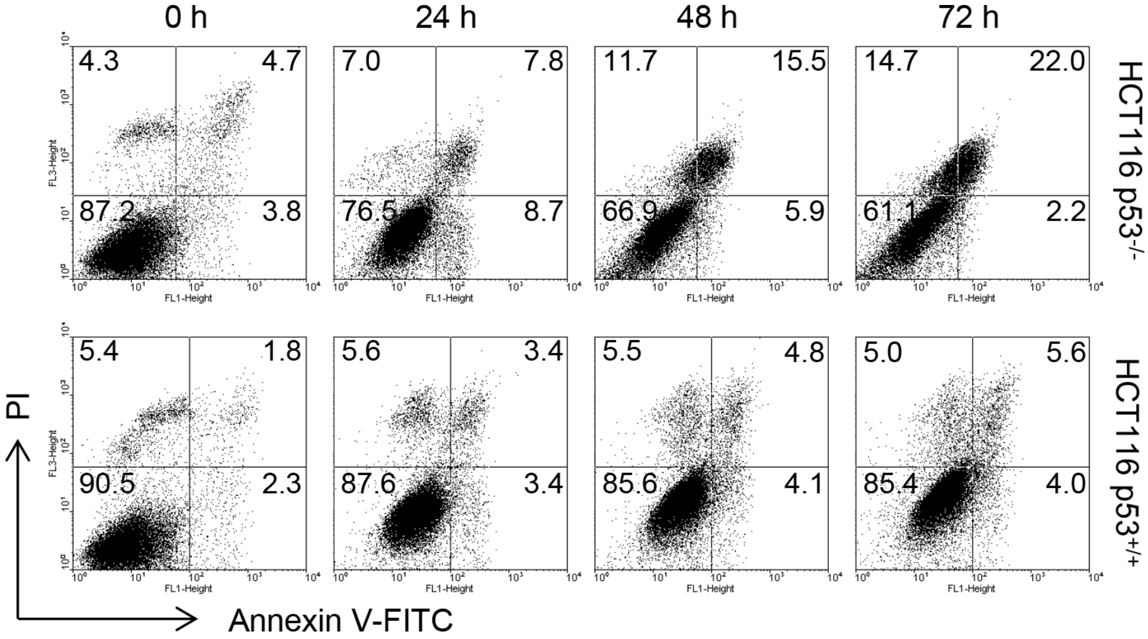

Supplementary Figure 1: C-1311 induces apoptosis in p53-deficient cells. HCT116 $p53^{+/+}$ and $p53^{-/-}$ cells were exposed to C-1311 (IC_{80} concentration - 0.68 μM for $p53^{+/+}$ and 0.64 μM for $p53^{-/-}$ cells) for the times indicated. The induction of apoptotic cell death was determined by FACS following Annexin V-FITC and propidium iodide (PI) staining. Lower left quadrant represents healthy cells (Annexin V-FITC-negative/PI-negative), lower right quadrant represents early apoptotic cells (Annexin V-FITC-positive/PI-negative), and upper right quadrant represents late apoptotic cells (Annexin V-FITC-positive/PI-positive). Data shown are representative of three independent experiments.

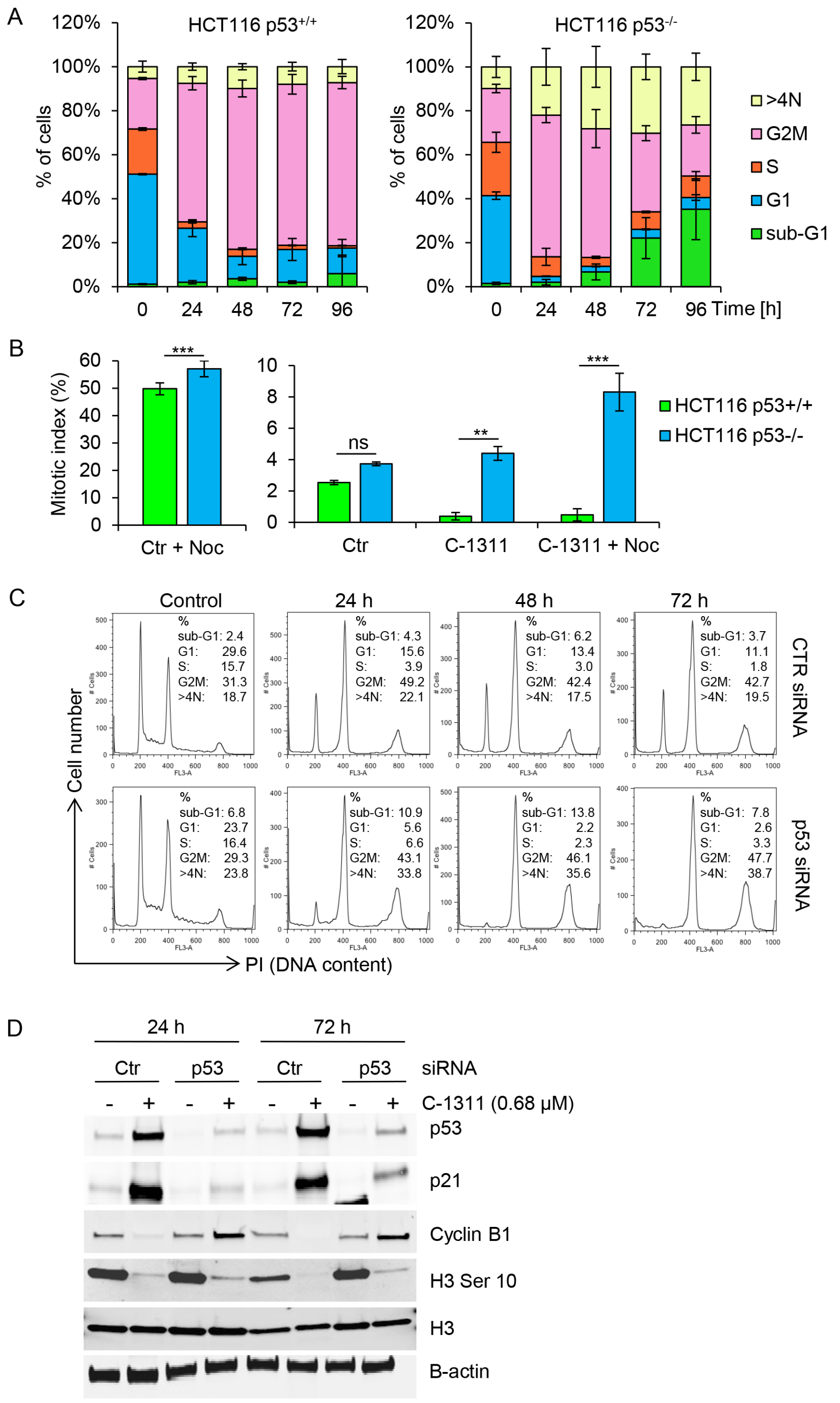

Supplementary Figure 2: C-1311 exerts a p53-dependent effect on the cell cycle. (**A**) The proportion of HCT116 p53$^{+/+}$ and p53$^{-/-}$ cells in different phases of the cell cycle following C-1311 exposure (IC80 concentration - 0.68 μM for p53$^{+/+}$ and 0.64 μM for p53$^{-/-}$ cells) as determined by FACS is shown. Results are a mean ± SD, n = 3. (**B**) HCT116 p53$^{+/+}$ and p53$^{-/-}$ cells were exposed to vehicle (Ctr) or C-1311 (IC80 concentration - 0.68 μM for p53$^{+/+}$ and 0.64 μM for p53$^{-/-}$ cells) for 24 h and then incubated with nocodazole (150 nM) for an additional 18 h. Cells were analyzed for mitotic index on the basis of the expression of histone H3 phosphorylated at Ser10 as quantified by FACS. Bars are the mean ± SD, n = 4. Significance: one-way ANOVA test with Bonferroni correction. $**P < 0.01$; $***P < 0.001$; ns, non-significant. (**C**) RKO cells were treated with p53 or scrambled (CTR) siRNA for 24 h, and then siRNA was removed and cells were exposed to C-1311 (0.68 μM) for the times indicated. DNA content analysis of cells stained with PI as quantified by FACS. Histograms are representative of three experiments. (**D**) Western blotting of RKO cells from part (C). p53, p21, cyclin B1, H3 and H3-Ser10 antibodies were used. β-actin was used as a loading control.

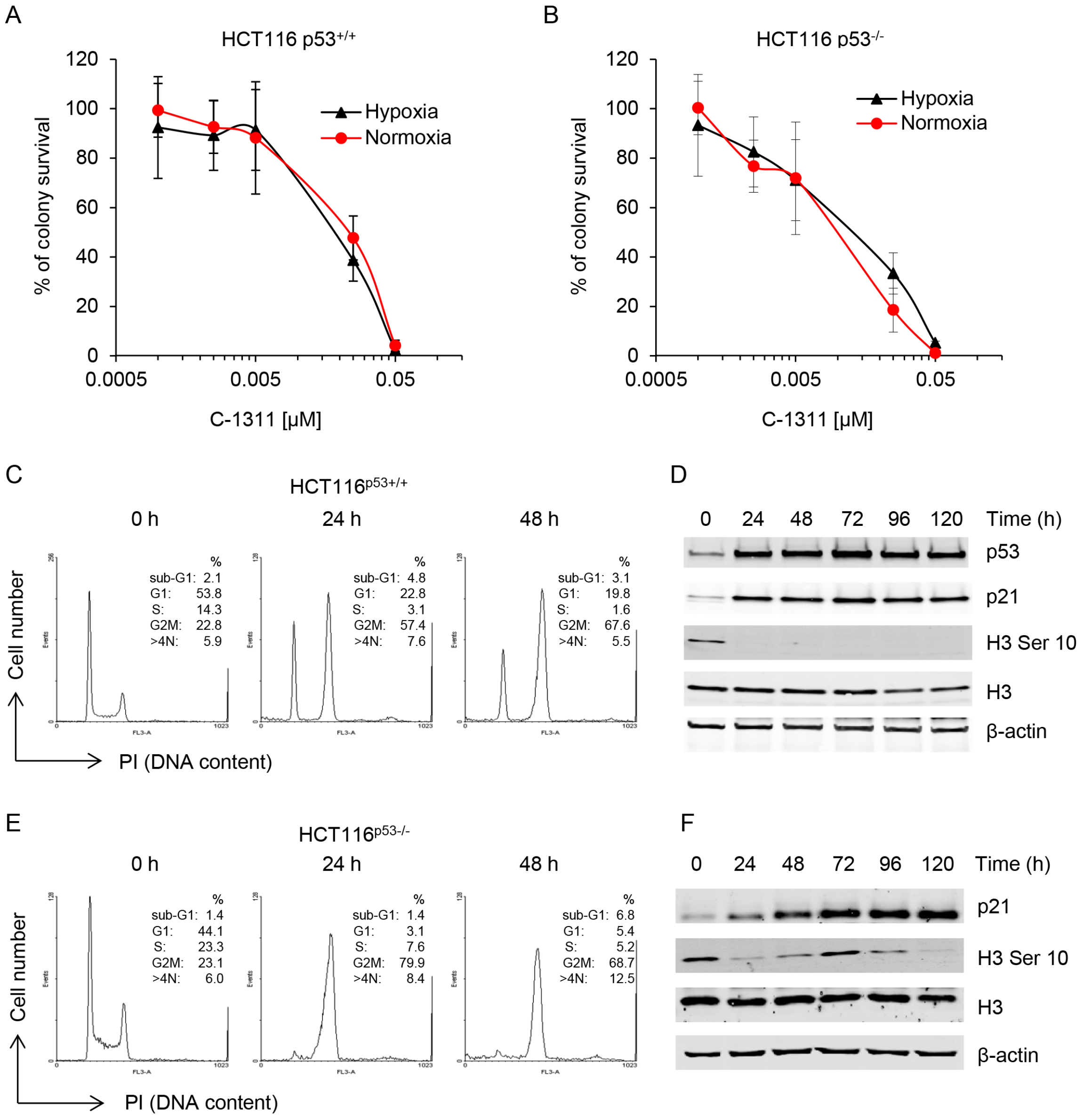

Supplementary Figure 3: C-1311 sustains its activity under physiologically relevant conditions. (**A**, **B**) HCT116 $p53^{+/+}$ and $p53^{-/-}$ cells were exposed to vehicle or a range of doses of C-1311 under normoxia (21% O^2) or hypoxia (1% O^2). After 72 h cells were transferred to normoxic conditions and colonies allowed to form, which were then counted after 14 days to determine the survival fraction. Results are a mean ± SD, $n = 3$. (**C, E**) HCT116 $p53^{+/+}$ and $p53^{-/-}$ cells were exposed to C-1311 (0.68 µM for $p53^{+/+}$ and 0.64 µM for $p53^{-/-}$ cell) under hypoxia (1% O_2), and DNA content was determined following PI staining and FACS. The 0 h time point indicates untreated cells incubated under hypoxia for 24 h. Results were analyzed using WinMDI software. Histograms are representative of three independent experiments. (**D**, **F**) Western blotting analysis of p53, p21, cyclin B1, H3 and H3-Ser10 in HCT116 $p53^{+/+}$ and $p53^{-/-}$ cells. Cells were exposed to C-1311 and hypoxia as in (C and E). β-actin was used as a loading control.

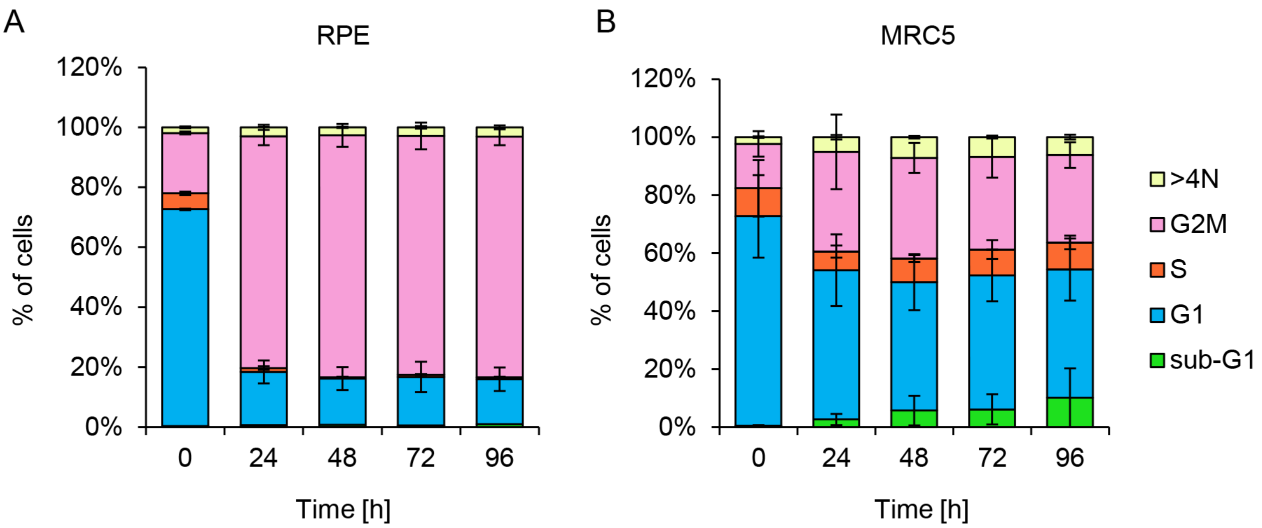

Supplementary Figure 4: Effect of C-1311 on cell cycle of non-cancer cells. (**A**, **B**) The proportion of retinal pigment epithelial RPE cells (A) and fetal lung fibroblast MRC5 cells (B) in different phases of the cell cycle following C-1311 exposure (0.68 µM) as determined by FACS. Results are a mean ± SD, $n = 3$.

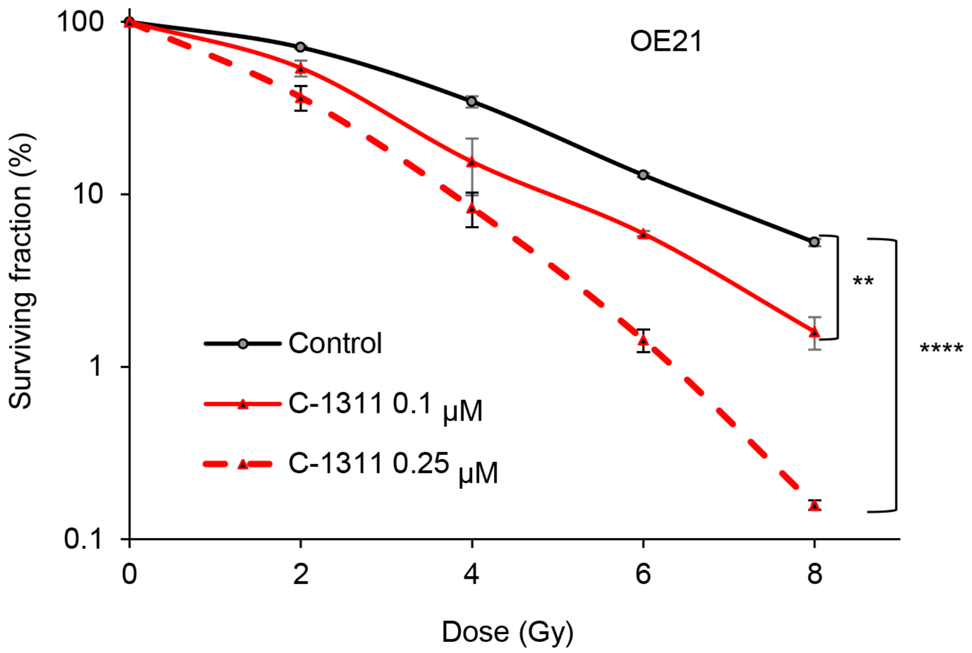

Supplementary Figure 5: Dose-dependent effect of C-1311 on sensitizing esophageal OE21 cells to radiation. Cells were treated with C-1311 (0.1 µM and 0.25 µM) for 1 hour before exposure to a range of doses of ionizing radiation (0, 2, 4, 6 and 8 Gy). Clonogenic assays were then carried out. Results shown are mean ± SEM, $n = 3$. Significance: Two-way ANOVA test,** $P < 0.01$; **** $P < 0.0001$.

Supplementary Table 1: Comparison of survival fraction at the radiation dose 2 Gy (SF_2)

Cell line	p53 status	SF_2 (%) Control	SF_2 (%) C-1311 (0.1 µM)
$HCT116^{p53+/+}$	wild-type	41.21 ± 4.9	27.51 ± 1.2
$HCT116^{p53-/-}$	null	48.04 ± 2.9	26.64 ± 5.4
OE21	mutated	71.12 ± 1.4	53.99 ± 5.7
FLO1	mutated	49.62 ± 1.3	25.82 ± 1.9
H1299	null	63.45 ± 9.0	21.28 ± 6.9

Results are a mean ± SEM, n = 3.
